# Role of α-Helical Structure in Organic Solvent-Activated Homodimer of Elastase Strain K

**DOI:** 10.3390/ijms12095797

**Published:** 2011-09-09

**Authors:** Raja Noor Zaliha Raja Abd. Rahman, Abu Bakar Salleh, Mahiran Basri, Chee Fah Wong

**Affiliations:** 1 Enzyme and Microbial Technology Laboratory, Faculty of Biotechnology and Biomolecular Sciences, Universiti Putra Malaysia, UPM Serdang 43400, Selangor, Malaysia; E-Mails: abubakar@biotech.upm.edu.my (A.B.S.); chee_fah_2000@yahoo.com (C.F.W.); 2 Faculty of Science, Universiti Putra Malaysia, UPM Serdang 43400, Selangor, Malaysia; E-Mail: mahiran@science.upm.edu.my

**Keywords:** *P. aeruginosa* strain K, organic solvent tolerant protease, dimerization, secondary structures

## Abstract

Recombinant elastase strain K overexpressed from *E. coli* KRX/pCon2(3) was purified to homogeneity by a combination of hydrophobic interaction chromatography and ion exchange chromatography, with a final yield of 48% and a 25-fold increase in specific activity. The purified protein had exhibited a first ever reported homodimer size of 65 kDa by SDS-PAGE and MALDI-TOF, a size which is totally distinct from that of typically reported 33 kDa monomer from *P. aeruginosa*. The organic solvent stability experiment had demonstrated a stability pattern which completely opposed the rules laid out in previous reports in which activity stability and enhancement were observed in hydrophilic organic solvents such as DMSO, methanol, ethanol and 1-propanol. The high stability and enhancement of the enzyme in hydrophilic solvents were explained from the view of alteration in secondary structures. Elastinolytic activation and stability were observed in 25 and 50% of methanol, respectively, despite slight reduction in α-helical structure caused upon the addition of the solvent. Further characterization experiments had postulated great stability and enhancement of elastase strain K in broad range of temperatures, pHs, metal ions, surfactants, denaturing agents and substrate specificity, indicating its potential application in detergent formulation.

## 1. Introduction

Enzymology in aqueous media has led to a molecular understanding of function of proteins over 100 years. In contrast to aqueous media, the term non-conventional media or nonaqueous enzymology refers to systems that use solvents other than water or the addition of components to aqueous systems with the intention of favoring specific properties of the biocatalyst or the reaction catalyzed by it [1]. Denaturation and inactivation of enzymes as the main nuisance in employment of organic solvents as the media for enzymatic reactions were no longer applicable when the breakthrough that enzymes especially proteases can be active and stabilized in organic solvents [2–5] had greatly expanded their potential for use in the syntheses of useful products [6]. In recent years, homogeneous non-aqueous enzymology emerges as an attractive alternative to other non-aqueous systems in order to overcome some inherent drawbacks associated with heterogeneous non-aqueous biocatalysis [7]. The use of circular dichroism (CD) spectroscopy, on the other hand, has directed to a better understanding of protein conformation in these media in regards to its secondary structures changes [8,9].

Along with this development, an extracellular organic solvent tolerant protease producer, *Pseudomonas aeruginosa* strain K, was isolated from benzene-toluene-ethylbenzene-xylene (BTEX) and polycyclic-aromatic-hydrocarbons (PAHs) compounds [10]. The formulations of physical and nutritional factors affecting the enzymes production, recently, have led to the optimized and bulk production of proteases from *P. aeruginosa* strain K [11,12]. Due to an overwhelming interest in exploring the organic solvent tolerant property possessed by the bacterium, its wild-type proteases were successfully purified and well characterized to withstand in numerous organic solvents of both hydrophilic and hydrophobic nature [13,14]. In this communication, our effort is focused on the characterization of recombinant elastase strain K which attributed hugely on the effect of organic solvents on the protein elastinolytic activity in relation to its structure.

## 2. Results and Discussion

### 2.1. Purification of Elastase Strain K

The accomplishment of the HIC objectives as the capture phase was clearly evidenced on Table 1. This was proven by an 11-fold increase in specific activity in addition to succession of 89% in yield (Table 1). The advantage of IEX in purifying proteins is that most of the proteins remain bioactive [15]. Therefore, a great increase in purification fold accompanied by low loss of protein (yield) is among the successes in this purification. The perfect combination of HIC and IEX had resulted in a 25-fold increase in specific activity against the crude (Table 1). Despite the employment of two chromatographic processes, this series of liquid chromatography had efficiently retained a final yield of 48% (Table 1). In some instances, HIC and IEX were reviewed to play a major role in protein folding [16].

### 2.2. Determination of Molecular Mass

**Figure 1:**
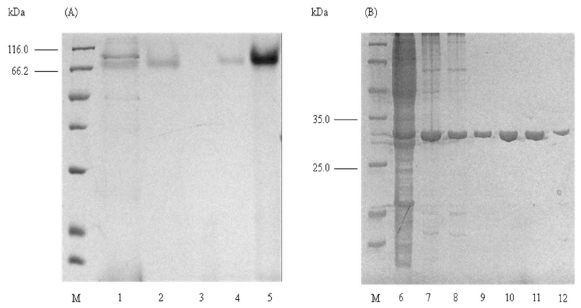
Electrophoresis of elastase strain K on non-reducing SDS-PAGE. (**A**) Samples without TCA precipitation; (**B**) Samples with TCA precipitation. Estimation of molecular weight is assisted by Unstained Protein Molecular Weight Marker (Fermentas, USA) in lane M. Abbreviation: crude, lane 1, 6; HIC, lane 2, 7; buffer exchange, lane 3, 8; IEX, lane 4, 9, 10, 11, 12; concentrated protein after IEX, lane 5.

Initial investigation into the molecular weight of elastase strain K was conducted by means of size-based separation of molecules in non-reducing SDS-PAGE, both with and without the pre-treatment of TCA. It was surprising that preliminary observation of non-TCA precipitated sample, which represented the native form of the protein, on the SDS-PAGE had unveiled a size of approximately 66 kDa (Figure 1A) It is twice as large as the size of wild-type elastase strain K [14] and other reported purified elastases from *P. aeruginosa*, which are estimated to be 33 kDa [4,6,17,18]. These untreated proteins, ranging from crude, HIC, buffer exchange, IEX and concentrated sample after IEX, had exhibited similar sizes of 66 kDa (Figure 1A).

A reduction in size to its reported 33 kDa was observed upon pre-treatment of proteins with TCA. Similarly, the size of precipitated proteins from crude, HIC, buffer exchange and IEX were examined to be at approximately 33 kDa (Figure 1B). Therefore, these preliminary results may provide an indication that the recombinant elastase strain K is expressed as dimers in the periplasm of *E. coli* KRX/pCon2(3). Conformational rearrangement of the protein structures may be involved in the dimerization and oligomerization processes [19]. Here, it was evidenced that the action by TCA, which acted as a weak carboxylic acid, on native protein had diminished the linkages between the monomers to return the size from 66 to 33 kDa. The appearance of dimeric form of this protein is probably contributed by noncovalent interactions as polar interactions between electronegative atoms, donors and acceptors, for example in hydrogen bond and close proximity interaction between oppositely charged atoms(<4.0 Å apart) in salt bridges [20,21] are most likely to occur in elastase strain K. The disulphide bond, on the other hand, is unlikely to cause the formation of dimer as the structure of elastase from *P. aeruginosa* is reported to be linked by Cys30 and Cys270 to Cys58 and Cys297, respectively [22].

### 2.3. Matrix-Assisted Laser Desorption/Ionization Time-of-Flight/Time-of-Flight (MALDI ToF/ToF) Mass Spectrometry

**Figure 2:**
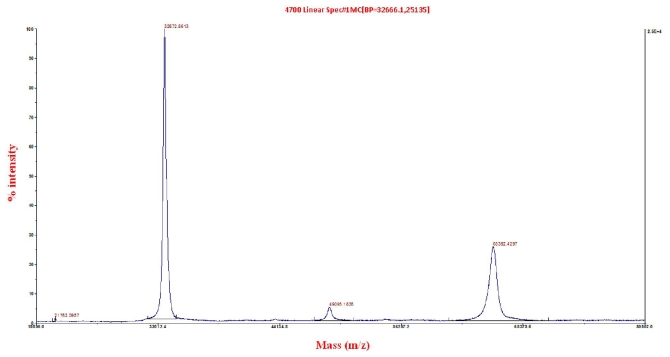
Separation profile of elastase strain K in MALDI-ToF/ToF.

In general, the SDS-PAGE systems do not always provide accurate molecular mass [23]. Thus, the purified native elastase strain K was subjected to quantitative analysis by MALDI ToF/ToF for determination of molecular weight. Two major peaks, each at 32,673 and 66,392 Da, can be seen in Figure 2. The structural conformation of elastase strain K as dimer was further confirmed from the fact that the peak of 32,673 Da which represented the monomer was derived as a result of linkages breakage by sinapinic acid on 66,392 Da dimer (which mimicked the action of TCA as discussed earlier) prior to protein spotting on MALDI target plate. The lower peak of 66,392 might be present as remaining ‘uncut’ dimers of the enzyme. In addition, protein identification search using NCBInr had depicted a confidence interval (C.I%) score of 100% with elastases from *P. aeruginosa* such as gi|52695999, gi|151212, gi|15598919, gi|116051721, gi|154127045, gi|194553400 and gi|152985549 (supplementary information 1).

### 2.4. Native PAGE and Activity Staining

**Figure 3:**
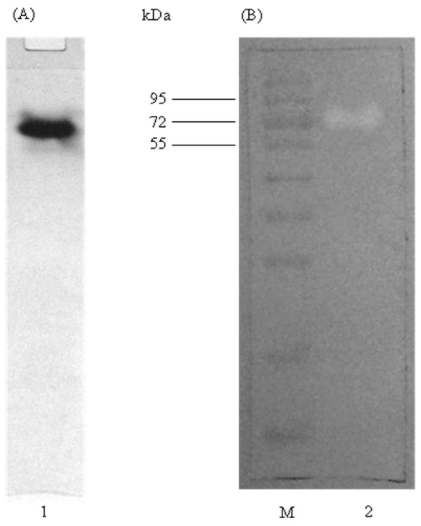
Conformation of purity and activity of elastase strain K. (**A**) Native PAGE; (**B**) Activity staining. Purified proteins after IEX were loaded into lane 1 and 2. PageRuler™ Prestained Protein Ladder (Fermentas, USA) is represented by lane M.

The purity of elastase strain K after HIC and IEX was examined by native PAGE. Native PAGE allows separation and recovery of native (non-denatured) proteins, which are more suitable for functional studies and for immunization [23]. Figure 3A had clearly shown that elastase strain K was purified to homogeneity as only a single band was observed on the gel. Furthermore, the protein still remained bioactive after purification as indicated by the presence of hydrolysis zone on skim milk agar in activity staining (Figure 3B).

### 2.5. Characterization of Elastase Strain K

Characteristics of the purified recombinant elastase strain K are summarized in Table 2.

#### 2.5.1. Effect of Temperatures on Enzyme Activity and Stability

The activity of elastase strain K had increased gradually from 25–37 °C, at relative activities of 91–100%. Upon reaching 40 °C, the elastinolytic activity had achieved its optimum relative point of 104%. The activities at 45, 50 and 55 °C, on the other hand, were observed to be stabilized at relative activities of 97, 96 and 95%, respectively. Further increase in temperature had resulted in decline of the elastinolytic activities, as viewed on 60, 65, 70, 75 and 80 °C. The highly regarded thermal stability of the enzyme was observed as relative activities of the protein were able to retain at above 80% in 4, 25, 30, 37, 40, 45, 50, 55 and 60 °C. At 70 °C, the relative activity had declined to 5% and totally diminished at 75 and 80 °C. The half-life of elastase strain K was estimated to be at 112 and 30 min in 55 and 65 °C, respectively.

#### 2.5.2. Effect of pH on Enzyme Activity and Stability

The activity was started low at 1 and 16% at a pH of 4 and 5, respectively, followed by a sharp elevation to 100% in an optimum pH of 6. Further increase in pH values had resulted in gradual fall of activity with total loss of elastinolytic activity was experienced in pH 10, 11 and 12. This result had indicated that elastase strain K prefers a slightly acidic environment for catalysis. The pH stability of elastase strain K, on the other hand, was resided in a broad pH range of 5–11.

#### 2.5.3. Effect of Additional Metal Ions on Enzyme Stability

The presence of most of the chloride metal ions tested at 5 and 10 mM had basically conferred no significant effect on the stability of elastase strain K as relative stabilities between 85–95% were discovered among Na^+^, K^+^, Mg^2+^, Ca^2+^, Mn^2+^, Co^2+^ and Sr^2+^. The presence of Cu^2+^ and Fe^3+^ at 10 mM, in contrast, had recorded greater falls of 50 and 93% from its original stability, respectively. The detrimental behaviour of Ni^2+^ and Zn^2+^ on elastase strain K was clearly seen at 10 mM since the relative stabilities were observed at merely 12 and 1%, respectively.

#### 2.5.4. Effect of Inhibitors on Enzyme Stability

A sharp fall of relative stability was discovered at 4 and 32% upon addition of respective metalloprotease inhibitors, 1,10-phenanthroline and ethylenediaminetetraacetic acid (EDTA), at 5 mM to the enzyme solution. It was not surprising to note that the presence of 1,10-phenantroline and EDTA at 10 mM had resulted in total wipe out of activities to 0 and 5%, respectively, since the presence of both of the inhibitors at 5 mM was enough to inhibit the activity of elastase strain K. The elastase strain K is, therefore, categorized as a metalloprotease based on the inhibitory effect exhibited by 1,10-phenanthroline and EDTA in both concentrations. In contrary, phenylmethylsulfonyl fluoride (PMSF), pepstatin A and antipain which each represented the inhibitor for serine, aspartic and cyteine proteases, did not affect the enzyme as relative activity was observed at above 90%.

#### 2.5.5. Effect of Denaturing and Reducing Agents on Enzyme Stability

Stimulatory effect of elastase strain K was detected in surfactants such as Triton-X-100 and Tween 20 at an increase of 22 and 5%, respectively, against its non surfactant containing enzyme solution (100%). The enzyme, alternatively, was also able to withstand the denaturing effect of 6 M urea that relative stability of 83% was discovered upon incubation at 37 °C for 30 min. The slight drop of stability in concentrated urea may be explained due to the strong interaction of water molecules with urea that water is likely to compete for internal hydrogen bonds of the protein, and thus initiate protein unfolding [24]. The incorporation of reducing agents especially DTT had indeed resulted in the significant lost of stability to an extent of approximately 99%. This observation had further recognized the presence of disulphide bonds that conferred structural and organic solvent stabilities in elastase strain K. Unlike Triton-X-100 and Tween 20, the presence of SDS had contributed to immediate decline in relative stability to 12%.

#### 2.5.6. Substrate Specificity

For liberation of products detected at 280 nm, casein, a major protein component in milk, was visualised to possess highest hydrolytic action by elastase strain K followed by haemoglobin and albumin (egg) with the absorbance of 1.8, 0.9 and 0.3, respectively. Azocasein, an azo-dyed derived-casein, was well hydrolysed by the enzyme with detection of 0.8 at 440 nm. Elastin Congo-red as the most specified substrate for elastases, had recorded liberation of its product at 0.2 in 495 nm. Hydrolysis of Azocoll, an azo dye suspended collagen, was detected at an absorbance reading of 0.6 at 510 nm.

#### 2.5.7. Effect of Organic Solvents on Enzyme Stability

**Figure 4:**
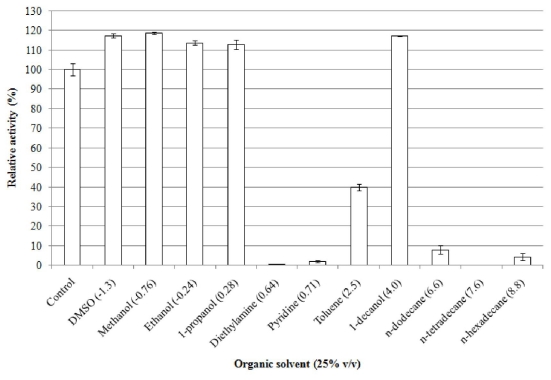
Stability of elastase strain K in the presence of 25% (v/v) organic solvents. Log *P*_o/w_ value for each organic solvent is stated in brackets. Elastinolytic activity in aqueous solution (without organic solvent) is regarded as control (100%). Relative activities are represented by mean value ± standard deviations (*n* = 3). The absence of a bar indicates that errors were smaller than the bars.

Analysis of the effect of organic solvents on the stability of elastase strain K had depicted that this enzyme is able to withstand in most of the organic solvents such as dimethylsulfoxide (DMSO), methanol, ethanol, 1-propanol, toluene and 1-decanol with *P*_o/w_ of −1.3, −0.76, −0.24, 0.28, 2.5 and 4.0, respectively, upon incubation at 37 °C for 30 min (Figure 4). Activity stabilities were not only observed on organic solvents with log *P*_o/w_ < 2 (DMSO, methanol, ethanol and 1-propanol), in fact, enhancement of activity was also observed in these water miscible organic solvents. It had been noted that DMSO, for instance, had enhanced the activity elastase strain K of approximately 17% followed by 19, 14, and 13%, each by methanol, ethanol and 1-propanol, compared to their non organic solvent containing-control. The enzyme, however, had experienced severe reduction in respect to its stability in other organic solvents such as diethylamine and pyridine with remaining activities detected only at 1 and 2%, respectively. Toluene, an organic solvent with *P*_o/w_ between 2.0–4.0, on the other hand, retained the elastinolytic activity of up to 40%. General observation on organic solvents with log *P*_o/w_ > 4 had deduced that majority of the tested solvents in this category had nearly diminished the activity of elastase strain K. Exception was recognized on 1-decanol of having *P*_o/w_ equivalent to 4.0 whereby this organic solvent had assisted in enhancement of relative activity to 117.0%. Unlike in the latter solvent, the enzyme was unable to display stability in *n*-dodecane and *n*-hexadecane with respective log *P*_o/w_ of 6.6 and 8.8 as activities were only observed at 8 and 4%, respectively. Additionally, *n*-tetradecane had posed as the most detrimental organic solvent to an extent that no detectable remaining activity observed for elastase strain K. The instability of enzymes in water immiscible organic solvents can be elucidated by the effect of phase toxicity resulted by the presence of separate organic phase. The effect of phase toxicity depends on both the nature of the water organic interface and the interfacial area; a large interfacial area may contribute to an increase in enzymatic inactivation [25].

The toxicity of an organic solvent correlates negatively with the parameter log *P*_o/w_ [26]. The results exhibited by the elastase strain K, however, contradicts with the latter rule as the ionization state of the protein is not taken into account in the log *P*_o/w_ model [26]. This can be explained by the capability of the hydrophilic organic solvents to mimic the effect of water and thereby preserve their enzyme structure [27]. This statement was supported by [28], in which the activity of subtilisin was only detected in hydrophilic solvents such as glycerol, ethylene glycol and 1,3-propanediol. A study carried out with lysozyme dissolved also in hydrophilic solvents such as in DMSO, DMF, formamide, methanol and ethylene glycol suggested that the retention of native protein structure is favored in very hydrophilic solvents with strong hydrogen bonding propensities [29]. With exception to activity enhancement in *n*-hexane and *n*-dodecane, the wild type of elastase strain K had projected the similar organic solvent stability profiles as its recombinant form [14]. The stability of enzymes in organic solvents usually stems from the nature of its protein structures and conformations such as retention of secondary structures [30,31], amount of α-helix and β-sheet [31], conformational change of amino acid in the active site [32], presence of intramolecular disulphide linkages [33] and presence of several surface-located amino acid residues to prevent the penetration of organic solvents into the interior of the protein molecule [34].

#### 2.5.8. Effect of Methanol on Enzyme Activity and Structure

Methanol, being the solvent which conferred high stability and activity enhancement to the enzyme (Figure 4), was further examined to determine the effect of the solvent concentrations on protease activity (Table 3). It was generally noted that the enzyme was capable of retaining the activity at concentrations as high as 50% (v/v). As expected, the elastinolytic activity of elastase strain K was improved by 15% compared to its non-methanol containing-control of 100%, upon addition of 25% (v/v) of methanol to the enzyme solution. Slight fall on the enzyme stability was observed in 50% (v/v) of methanol. The stability, however, was somehow maintained at 98% in this particular concentration. In contrast, the elastase strain K underwent a sharp decline in relative stability following incubation with 75% (v/v) of the solvent, resulting in detection of 30% in relative activity. Subsequent increase in concentration of methanol to 90% (v/v) had attributed to destructive effect on the enzyme stability where the remaining activity uncovered was only at 4%. A similar stability trend was observed for *B. licheniformis* S-85 esterase that higher enzymatic activity was detected at 25–50% glycerol followed by great reduction in concentrations of more than 65% [7].

The stability of elastase strain K in methanol can be explained by using glycerol as the model. Similar to other hydrophilic organic solvents, glycerol is capable of mimicking the effect of water and thereby preserving enzyme structure [27]. Owing to its properties, glycerol is one of the most studied organic solvents in homogeneous solvent mixtures. The dielectric constant of glycerol (~47.0) is approximately half that of water (~88.0) and is therefore not low enough, compared to the natural organic solvents, to increase the rigidity of the molecules (due to strong intra-protein interactions), nor high enough to destabilize the tertiary structure of the proteins as other organic solvents do [35,36]. Motions in proteins are governed mostly by electrostatic interactions [32] and are particularly influenced by the dielectric constant of the solvent, with an increasing solvent dielectric constant, the motions in proteins become more rapid [36]. Therefore, methanol, having a dielectric constant of ~40.0 (which also approximately half to that of water), might share analogous behavior and effect of glycerol towards enzymes.

The secondary structures of elastase strain K in 0, 25, 50, 75 and 90% (v/v) of methanol were relatively estimated using CONTINLL curve fitting in DICHROWEB. In the determination of secondary structures, both α-helix and β-sheet were sub-categorized into regular and distorted fractions. The secondary classes of distorted α-helix and β-sheet were formed by considering 1–3 and 1–4 residues for each end of α-helix and β-sheet segments, respectively, to be distorted, thus producing CD signals that are different from its regular segments [37]. It can be generally seen from Table 4 that the regular and distorted α-helix contents had experienced gradual decreased upon transition from 0–50% (v/v) of methanol, followed by rapid decline in 75 and 90% (v/v). Additionally, the presence of 90% (v/v) methanol contributed to the total loss of regular α-helix structure. In contrast, the unordered forms of protein which include random coil had gradually increased following incorporation of higher concentrations, indicating that most of the α-helices were unfolded to form non-typical structures (random). The regular and distorted β-sheets as well as turn, on the other hand, were reported to be stabilised throughout the transitions in CONTINLL.

Based on the data from protease activity and spectral patterns generated by the various concentrations of methanol, it was well distinguished that the changes in secondary structure contents correlate with the elastinolytic activities exhibited in each concentration of methanol and made explainable in two ways. First, the amino acid residues proposed to involve in the catalysis of a three dimensional structure of elastase, Glu141, Tyr155 and His223, are located at distorted and regular segments of the α-helix [22]. Although the reduction of these regions was observed throughout the experiment, some parts in the α-helical structure in which these residues might be resided, were somehow preserved and thereby resulted in recovery of activities in 25–50% (v/v) of methanol. Further increase in methanol concentrations had led to severe rupture of this region along with loss of activity. The second explaination stems from the stability of poly-L-glutamic acid and poly-L-proline that formed the helical structures. It was shown that the CD spectra of these polyamino acids were unchanged in the presence of 25% (v/v) methanol [31]. Thus, similar hypothesis might be applied on the structure of elastase strain K, in which enhancement of activity was also seen in this particular concentration. The presence of methanol in high concentrations, on the other hand, had rendered the rule unapplicable as distruption of α-helical structures were visualised in these conditions (75–90%).

## 3. Experimental Section

### 3.1. Preparation of Crude Elastase Strain K for Protein Purification

An overnight culture of *E. coli* KRX/pCon2(3) (1% v/v) was inoculated into 500 mL shake flask containing 50 mL of LB broth, supplemented with carbenicillin (50 μg/mL). Based on the optimization studies, the bacterial cells were further propagated up to *A_600nm_* = 0.75 prior to induction with 0.2 mM of IPTG for 8 h at 200 rpm and 37 °C. The recombinant cells were centrifuged at 10,000 rpm for 10 min at 4 °C and the pellet was subsequently resuspended with an equal volume of 50 mM Tris-Cl (pH 8.0), 1.0 M (NH_4_)_2_SO_4_. Sonication was performed 3 times at an intermittent of 30 s with control output and duty cycle of 8 and 30%, respectively. The soluble protein was obtained after centrifugation at 10,000 rpm for 10 min at 4 °C.

### 3.2. Hydrophobic Interaction Chromatography (HIC)

The crude recombinant elastase strain K was loaded into a XK16 column (GE Healthcare, Sweden) contining 5 mL Butyl-S Sepharose Fast Flow (GE Healthcare, Sweden) pre-equilibrated with 50 mM Tris-Cl (pH 8.0), 1.0 M (NH_4_)_2_SO_4_ at a flow rate of 1 mL/min. The unbound proteins were eliminated using 7 column volume (CV) of 50 mM Tris-Cl (pH 8.0), 1.0 M (NH_4_)_2_SO_4_ and bound proteins were eluted with 20 CV of descending linear gradients of (NH_4_)_2_SO_4_ ranging from 1 to 0 M at a flow rate of 1 mL/min. Fractions (2.5 mL) were collected and assayed for elastinolytic activity [38]. Positive fractions were pooled for subsequent overnight dialysis against 5 L of 50 mM Tris-Cl (pH 8.5) at 4 °C.

### 3.3. Ion Exchange Chromatography (IEX)

With a flow rate of 1 mL/min, the dialyzed protein sample was injected into XK16 column containing 5 mL of Q-Sepharose Fast Flow (GE Healthcare, Sweden), pre-equilibrated with 50 mM Tris-Cl (pH 8.5), as binding buffer. The column was then washed with 7 CV of the binding buffer followed by ascending linear gradient elution with 20 CV of NaCl varying from 0 to 0.5 M. Eluate size was set at 2.5 mL per fraction, in which all fractions were subjected to elastinolytic assay [38]. Similarly, fractions with elastinolytic activities were pooled and removal of salts was performed by overnight dialysis against 50 mM Tris-Cl (pH 8.5) at 4 °C.

### 3.4. Characterization of Recombinant Elastase Strain K

The activity and stability of recombinant elastase strain K in each tested parameters were measured according to elastinolytic assay, as outlined by [38].

#### 3.4.1. Matrix-Assisted Laser Desorption/Ionization Time-of-Flight/Time-of-Flight (MALDI ToF/ToF)

Mass Spectrometry Analysis Mass spectra of recombinant elastase strain K were acquired on ABI 4800 Proteomics Analyzer MALDI TOF/TOF™ mass spectrometer (Applied Biosystems, USA), in which the entire experiment was carried out by First Base Ptd., Singapore. Briefly, the protein was mixed with same amount of matrix solution containing 10 mg/mL of 3, 5-dimethoxy-4-hydroxy cinnamic acid (sinapinic acid) in 0.1% trifluoroacetic acid (TFA)/50% acetonitrile (ACN), and spotted onto a 384-well stainless-steel MALDI target plate (Applied Biosystems, USA). A total of 1200 laser shots were accumulated for each spectrum with laser intensity of 5000. All mass spectra data was analyzed using 4000 Series Explorer™ software (Applied Biosystems, USA).

#### 3.4.2. Sodium Dodecyl Sulfate Polyacrylamide Gel Electrophoresis (SDS-PAGE)

Sample preparation and protein electrophoresis by SDS-PAGE [39] was performed in non-reducing condition (without boiling and pre-treatment of β-mercaptoethanol). Pre-treatment of trichloroacetic acid (TCA) by means of TCA precipitation [40] and without the pre-treatment was carried out on protein samples. In TCA precipitation, the protein was subjected to TCA (100% (w/v)) at 10:1 ratio (% v/v) followed by 10 min of incubation on ice. The mixture was centrifuged at 10000 rpm for 10 min to obtain the precipitated protein. Washing step was carried out by Tris-Cl (50 mM, pH 8.5) with subsequent centrifugation at similar speed and duration. Sample without pre-treatment of TCA was loaded into native PAGE for visualization of the native form of protein.

#### 3.4.3. Activity Staining

Polyacrylamide gel containing purified recombinant elastase strain K and standard protein marker, PageRuler™ Prestained Protein Ladder (Fermentas, Canada), from the non-reducing SDS-PAGE was subjected to immersion in 20% (v/v) isoproponol to remove the SDS, with subsequent washing using ultrapure water (2–3 times) before being transferred onto SMA supplemented with carbenicillin (100 μg/mL). Proteolytic activity of the active protein was examined based on the existence of clearing zone at position of migrated protein in the polyacrylamide gel after 30 min of incubation at 37 °C.

#### 3.4.4. Effect of Temperature on Elastinolytic Activity

Investigation on hydrolysis of elastin Congo-red by recombinant elastase strain K in temperatures ranging from 25 to 80 °C with intervals of 5 °C was conducted in a shaking rate of 200 rpm for 30 min. Elastinolytic activities exhibited at the various temperatures were compared in relative to activity at 37 °C, which served as control (100%). In thermal stability test, the protein was pre-incubated in temperatures starting from 4 °C to 80 °C for 30 min in 200 rpm prior to elastinolytic assay. Half life of the enzyme was examined by pre-incubating the protein at 55 °C from 0–240 min in a waterbath shaker. Samples were removed at intervals of 30 min for measurement of activity, in which activity at 0 min is the control of the experiment (100%).

#### 3.4.5. Effect of pH on Elastinolytic Activity

The optimum pH for elastinolytic activity of recombinant elastase strain K was determined by the enzymatic hydrolysis of elastin Congo-red in tested 50 mM buffering systems of sodium acetate (pH 4–6), potassium phosphate (pH 6–8), Tris-Cl (pH 8–9), glycine-OH (pH 9–11) and sodium hydrogen phosphate (pH 11–12). In brief, the substrate was resuspended with buffers at various pHs and subsequently subjected to elastinolytic assay. Enzyme activity in 50 mM sodium acetate (pH 6) was further chosen as control for this experiment. In a pH stability test, the enzyme was pre-incubated with buffers of tested pHs at ratio of 1:3 (v/v) at 37 °C and 200 rpm for 30 min. It further underwent elastinolytic assay for verification of its pH stability.

#### 3.4.6. Effect of Additional Metal Ions on Elastinolytic Activity

In this study, the enzyme was pre-treated with 0, 5 and 10 mM of chloride (Cl^−^) metal ions such as Na^+^, K^+^, Mg^2+^, Ca^2+^, Mn^2+^, Co^2+^, Ni^2+^, Cu^2+^, Zn^2+^, Sr^2+^ and Fe^3+^ for 30 min at 37 °C before subjected to elastinolytic assay.

#### 3.4.7. Effect of Protease Inhibitors on Elastinolytic Activity

Protease inhibitors such as phenylmethylsulfonyl fluoride (PMSF), ethylenediaminetetraacetic acid (EDTA), *o*-phenanthroline, pepstatin A and antipain were used at final concentrations of 5 and 10 mM (with the exception of 1 and 2 mM for antipain) to treat the protein at 37 °C for 30 min. The inhibitor-free mixture was considered to be the control of experiment.

#### 3.4.8. Effect of Denaturing and Reducing Agents on Elastinolytic Activity

The effect of β-mercapthoethanol, Triton-X-100, Tween 20, SDS and dithiothreitol (DTT) at 0.5% (v/v) and urea (6 M) were tested on recombinant elastase strain K for 30 min at 37 °C. Elastinolytic assay was conducted to determine the residual activity, as compared to control (in the absence of denaturing and reducing agents).

#### 3.4.9. Substrate Specificity of Recombinant Elastase Strain K

Substrate preference of recombinant elastase strain K was monitored following hydrolysis of 0.5% (w/v) elastin Congo-red, casein, azocasein, haemoglobin, albumin (egg) and azocoll by the enzyme at 37 °C for 30 min. Dyed products, which were liberated from elastin Congo-red, azocasein and azocoll were measured colorimetrically at specific wavelength of 495, 440 and 510 nm, respectively. Soluble peptides and amino acids resulted from the digestion of casein, haemoglobin and albumin (egg) were detected at 280 nm [41].

#### 3.4.10. Organic Solvent Stability of Recombinant Elastase Strain K

The stability of recombinant elastase strain K in organic solvent was evaluated based on the remaining elastinolytic activity available after a pre-incubation of the enzyme:organic solvent (3:1 (v/v)) mixture at 37 °C with an agitation speed of 200 rpm for 30 min. Prior to pre-incubation, all final enzyme concentrations were adjusted to 0.1 mg/mL. Both water miscible and water immiscible organic solvents such as dimethylsulfoxide (DMSO), methanol, ethanol, 1-propanol, diethylamine, pyridine, toluene, 1-decanol, *n*-dodecane, *n*-hexadecane and *n*-tetradecane were tested on the enzyme. Control mixtures contained enzymes with no organic solvent being added. The residual activity was calculated based on relative activity obtained from enzyme-solvents mixtures toward control mixture and converted into the form of percentage. The control was recorded as 100%. Each batch mixture had its own control.

#### 3.4.11. Effect of Methanol Concentrations on Enzyme Stability

Purified proteins were prepared in a final concentration of 0.1 mg/mL in mixtures containing different concentrations of methanol ranging from 0, 10, 25, 50, 75 and 90% (v/v). Similar experimental protocols were carried out as stated at section 3.4.10. Enzyme stability (%) was expressed as the remaining elastinolytic activity in relative to the solvent-free (0% (v/v)) as the control (100%).

#### 3.4.12. Biophysical Characterization of Recombinant Elastase Strain K

Data of biophysical properties possessed by the protein were obtained from JASCO J-810 Circular Dichroism Spectropolarimeter (CD) (Tokyo, Japan). Purified protein in initial 50 mM Tris-Cl (pH 8.5) was buffer-exchanged to 20 mM sodium phosphate buffer (pH 7.0) using Sephadex G-25 at a constant flow rate of 1 mL/min. The protein was concentrated up to 2.7 mg/mL (to be kept as stock) via a 10.0 kDa molecular weight cut-off spin column (Amicon, Milipore, USA). By utilizing several parameters pre-programmed in Spectra Manager software (JASCO, Japan), the generated CD spectra can be used for analysis in secondary structure changes as well as thermal denaturation.

#### 3.4.13. Effect of Methanol on Protein Secondary Structure

The protein samples (0.1 mg/mL) were treated with various percentages of methanol ranging from 0, 25, 50, 75, 90% (v/v) at 37 °C for 30 min. Far-UV CD spectra were obtained at a region of 190–240 nm wavelength with 1.0 mm path length cuvette containing the native enzyme (0% methanol) and methanol-enzyme mixtures. Final spectra reading for each sample, calculated from an average of 5 scans, was baseline-corrected with their respective blank. Blank solutions were prepared by substituting the enzyme with buffer. The far-UV spectra were analyzed by an open source software, CONTINLL, by DICHROWEB [42,43] to ascertain the changes of secondary structures.

## 4. Conclusions

The discovery of a size of approximately 66 kDa in elastase strain K was evidenced as the first case of dimerization of elastase ever reported from *P. aeruginosa*. The organic solvent stability profile had indeed exhibited a contradictory trend in respect to the rules described by [26] whereby activity enhancement of elastase strain K was visualized in majority of organic solvents having log *P*_o/w_ < 2. Clear correlations were established between concentrations of methanol and changes in secondary structures of elastase strain K as enhancement and stability of the elastinolytic activities, as viewed on 25 and 50% of methanol, were explained by just the slight change of α-helical structures, presumably without disruption of active site. Sudden decrease of activity in 75 and 90% methanol, on the other hand, was described by the total loss of the helical structures followed by the increase in unordered form (random coil).

## Supplementary Information



## Figures and Tables

**Table 1:** Purification of elastase strain K from *E. coli* KRX/pCon2(3) by HIC and IEX.

Purification step	Volume (mL)	Activity (A_495_/h/mL)	Total activity (A_495_/h)	Protein content (mg/mL)	Total protein (mg)	Specific activity (A_495_/h/mg)	Yield (%)	Fold
**Crude**	50	7	368	0.5	23	16	100	1
**HIC**	22	15	326	0.1	2	181	89	11
**IEX**	6.6	27	177	0.1	0.4	403	48	25

**Table 2:** Effect of various parameters on the stability of recombinant elastase strain K.

Characterization	Characteristic	
Optimum temperature (°C)	40	
Thermal stability (°C)	4–60	
Optimum pH	6	
pH stability	5–11	
	**Relative activity (%)**	
	
	**5.0 mM**	**10.0 mM**
	
**Control**^a^	100	100
**Metal ion**		
Na^+^	94	99
K^+^	94	94
Mg^2+^	94	89
Ca^2+^	90	91
Mn^2+^	90	93
Co^2+^	90	87
Ni^2+^	17	12
Cu^2+^	81	55
Zn^2+^	30	1
Sr^2+^	92	94
Fe^3+^	82	7
**Protease inhibitor**		
PMSF	104	96
EDTA	32	5
*o*-phenanthroline	4	0
Pepstatin A	103	92
Antipain b	102	95
**Reducing and denaturing agent**^c^		
B-mercaptoethanol	83	
Triton-X-100	122	
Tween 20	105	
Urea (6 M)	83	
SDS	12	
DTT	1	
**Substrate specificity**	Casein, azocasein, elastin Congo-red, haemoglobin, egg albumin and Azocoll	

a Elastinolytic activities derived from non-treated enzyme solution are regarded as control;

b Subject was examined at concentrations of 1 and 2 mM;

c Enzyme was pre-incubated with 6 M urea and 0.5% (v/v) of other agents prior to elastinolytic assay.

**Table 3:** Stability of elastase strain K in various concentrations of methanol.

Concentration (% (v/v))	Relative activity (%) a
0	100 ± 4.52
25	115 ± 2.47
50	98 ± 1.44
75	30 ± 1.49
90	4 ± 0.13

a Relative activities are represented by mean value ± standard deviations (*n* = 3).

**Table 4:** Predicted secondary structures of elastase strain K dissolved in various concentrations of methanol as determined from far UV spectra.

Methanol (% (v/v))	α-helix a	α-helix b	β-sheet a	β-sheet b	Turn	Unordered
0	0.19	0.12	0.19	0.10	0.20	0.20
25	0.15	0.11	0.20	0.10	0.20	0.24
50	0.10	0.09	0.22	0.11	0.19	0.30
75	0.02	0.06	0.22	0.12	0.20	0.38
90	0.00	0.06	0.21	0.11	0.19	0.42

a Regular fraction;

b Distorted fraction.

## References

[1] Krieger N, Bhatnagar T, Baratti JC, Baron AM, de Lima VM, Mitchell D (2004). Non-aqueous biocatalysis in heterogeneous solvent systems. Food Technol Biotechnol.

[2] Ogino H, Watanabe F, Yamada M, Nakagawa S, Hirose T, Noguchi A, Yasuda M, Ishikawa H (1999). Purification and characterization of organic solvent-stable protease from organic solvent-tolerant *Pseudomonas aeruginosa* PST-01. J Biosci Bioeng.

[3] Karadzic I, Masui A, Fujiwara N (2004). Purification and characterization of a protease from *Pseudomonas aeruginosa* grown in cutting oil. J Biosci Bioeng.

[4] Doddapaneni KK, Tatineni R, Vellanki RN, Rachcha S, Anabrolu N, Narakuti V, Mangamoori LN (2009). Purification and characterization of a solvent and detergent-stable novel protease from *Bacillus cereus*. Microbiol Res.

[5] Rai SK, Mukherjee AK (2010). Statistical optimization of production, purification and industrial application of a laundry detergent and organic solvent-stable subtilisin-like serine protease (Alzwiprase) from *Bacillus subtilis* DM-04. Biochem Eng J.

[6] Ogino H, Yasui K, Shiotani T, Ishihara T, Ishikawa H (1995). Organic solvent stable-tolerant bacterium which secretes an organic solvent-stable proteolytic enzyme. Appl Environ Microbiol.

[7] Torres S, Castro GR (2004). Non-aqueous biocatalysis in homogeneous solvent systems. Food Technol Biotechnol.

[8] Menaa B, Menaa F, Aiolfi-Guimaraes C, Sharts O (2010). Silica-based nanoporous sol-gel glasses: from bioencapsulation to protein folding studies. Int J Nanotechnol.

[9] Menaa B, Montoneri C, Menaa F, Montoneri E, Boffa V, Sharts O (2011). Protein helical structure enhancement in biocompatible fluoro-phosphonate-based nanoporous silica glasses assessed by circular dichroism spectroscopy. Int J Nanotechnol.

[10] Geok LP, Razak CNA, Rahman RNZRA, Basri M, Salleh AB (2003). Isolation and screening of an extracellular organic solvent-tolerant protease producer. Biochem Eng J.

[11] Rahman RNZRA, Geok LP, Basri M, Salleh AB (2005). Physical factors affecting the production of organic solvent-tolerant protease by *Pseudomonas aeruginosa* strain K. Bioresour Technol.

[12] Rahman RNZRA, Geok LP, Basri M, Salleh AB (2005). An organic solvent-tolerant protease from *Pseudomonas aeruginosa* strain K: nutritional factors affecting protease production. Enzyme Microb Technol.

[13] Rahman RNZRA, Geok LP, Basri M, Salleh AB (2006). An organic solvent-stable alkaline protease from *Pseudomonas aeruginosa* strain K: Enzyme purification and characterization. Enzyme Microb Technol.

[14] Yusoff N (2007). Purification and Characterization of Organic Solvent Tolerant Protease from *Pseudomonas aeruginosa* Strain K. Master Thesis.

[15] Xindu G, Lili W (2008). Liquid chromatography of recombinant proteins and protein drugs. J Chromatogr B.

[16] Geng X, Wang C (2007). Protein folding liquid chromatography and its recent developments. J Chromatogr B.

[17] Cheng M, Takenaka S, Aoki S, Murakami S, Aoki K (2009). Purification and characterization of an eggshell membrane decomposing protease from *Pseudomonas aeruginosa* strain ME-4. J Biosci Bioeng.

[18] Lin X, Xu W, Huang K, Mei X, Liang Z, Li Z, Guo J, Luo Y (2009). Cloning, expression and characterization of recombinant elastase from *Pseudomonas aeruginosa* in *Pichia pastoris*. Protein Expr Purif.

[19] Li ZR, Liu GR, Cheng Y (2005). Thermodynamic analysis of protein sequence-structure relationships in monomer and dimer forms. Physica A.

[20] Jones S, Thornton JM (1995). Protein-protein interactions: a review of protein dimer structures. Prog Biophys Mol Biol.

[21] McKee T, JMcKee JR (2003). Enzymes.

[22] Thayer MM, Flaherty KM, McKay DB (1991). Three-dimensional structure of elastase of *Pseudomonas aeruginosa* at 1.5-Å resolution. J Biol Chem.

[23] Michalski WP, Shiell BJ (1999). Strategies for analysis of electrophoretically separated proteins and peptides. Anal Chim Acta.

[24] Schiffer CA, Dötsch V (1996). The role of protein-solvent interactions in protein unfolding. Curr Opin Biotechnol.

[25] Aldercreutz P, Koskinen AMP, Klibanov AM (1996). Modes of using enzymes in organic media. Enzymatic Reactions in Organic Media.

[26] Laane C, Boeren S, Vos K, Veeger C (1987). Rules for optimization of biocatalysis in organic solvents. Biotechnol Bioeng.

[27] Ru MT, Dordick JS, Reimer JA, Clark DS (1999). Optimizing the salt-induced activation of enzymes in organic solvents: Effects of lyophilization time and water content. Biotechnol Bioeng.

[28] Castro GR (1999). Enzymatic activities of proteases dissolved in organic solvents. Enzyme Microb Technol.

[29] Knubovets T, Osterhout JJ, Klibanov AM (1999). Strucutres of lysozyme dissolved in neat organic solvents as assessed by NMR and CD spectroscopies. Biotechnol Bioeng.

[30] Xu K, Griebenow K, Klibanov AM (1997). Correlation between catalytic activity and secondary structure of subtilisin dissolved in organic solvents. Biotechnol Bioeng.

[31] Ogino H, Gemba Y, Yutori Y, Doukyu N, Ishimi K, Ishikawa H (2007). Stabilities and conformational transitions of various proteases in the presence of an organic solvent. Biotechnol Prog.

[32] Sharma S, Tyagi R, Gupta MN, Singh TP (2001). Enhancement of catalytic activity of enzymes by heating in anhydrous organic solvents: 3D structure of a modified serine proteinase at high resolution. Indian J Biochem Biophys.

[33] Ogino H, Uchiho T, Yokoo J, Kobayashi R, Ichise R, Ishikawa H (2001). Role of intermolecular disulfide bonds of the organic solvent-stable PST-01 protease in its organic solvent stability. Appl Environ Microbiol.

[34] Ogino H, Uchiho T, Doukyu N, Yasuda M, Ishimi K, Ishikawa H (2007). Effect of exchange of amino acid residues of the surface region of the PST-01 protease on its organic solvent-stability. Biochem Biophys Res.

[35] Castro GR, Knubovets T (2003). Homogeneous biocatalysis in organic solvents and water-organic mixtures. Crit Rev Biotechnol.

[36] Affleck R, Haynes CA, Clark DS (1992). Solvent dielectric effects on protein dynamics. Proc Natl Acad Sci USA.

[37] Sreerama N, Venyaminov SY, Woody RW (1999). Estimation of the number of α-helical and β-strand segments in proteins using circular dichroism spectroscopy. Protein Sci.

[38] Ohman DE, Cryz SJ, Iglewski BH (1980). Isolation and characterization of a *Pseudomonas aerugionsa* PAO mutant that produces altered elastase. J Bacteriol.

[39] Laemmli UK (1970). Cleavage of structure protein during assembly of the head of bacteriophage T_4_. Nature.

[40] Bollag DM, Edelstein SJ (1991). Concentrating Protein Solutions.

[41] Cowan DA, Smolenski KA, Daniel RM, Mogan HW (1987). An extremely thermostable extracellular proteinase from a strain of the archaebacterium *Desulfurococcus* growing at 88 °C. Biochem J.

[42] Whitmore L, Wallace BA (2004). DICROWEB: an online server for protein secondary structure analyses from circular dichroism spectroscopic data. Nucleic Acids Res.

[43] Whitmore L, Wallace BA (2008). Protein secondary structure analyses from circular dichroisn spectroscopy: methods and reference databases. Biopolymers.

