# Supplementary material for: Role of α-Helical Structure in Organic Solvent-Activated Homodimer of Elastase Strain K
Source: Int J Mol Sci. 2011 Sep 9;12(9):5797–814. doi: 10.3390/ijms12095797 (PMC3189751; doi:10.3390/ijms12095797)
Supplement: Supplementary file 1 [file ijms-12-05797-s001.pdf]

Supplementary Information

Raja Noor Zaliha Raja Abd. Rahman [1,*], Abu Bakar Salleh [1], Mahiran Basri [2] and Chee Fah Wong [1]

[1] Enzyme and Microbial Technology Laboratory, Faculty of Biotechnology and Biomolecular Sciences, Universiti Putra Malaysia, UPM Serdang 43400, Selangor, Malaysia;
E-Mails: abubakar@biotech.upm.edu.my (A.B.S.); chee_fah_2000@yahoo.com (C.F.W.)

[2] Faculty of Science, Universiti Putra Malaysia, UPM Serdang 43400, Selangor, Malaysia;
E-Mail: mahiran@science.upm.edu.my

* Author to whom correspondence should be addressed; E-Mail: rnzaliha@biotech.upm.edu.my;
Tel.: +6-03-8946-6709; Fax: +6-03-8943-0913.

Received: 18 July 2011; in revised form: 5 August 2011 / Accepted: 11 August 2011 / Published: 9 September 2011

Abstract: Recombinant elastase strain K overexpressed from *E. coli* KRX/pCon2(3) was purified to homogeneity by a combination of hydrophobic interaction chromatography and ion exchange chromatography, with a final yield of 48% and a 25-fold increase in specific activity. The purified protein had exhibited a first ever reported homodimer size of 65 kDa by SDS-PAGE and MALDI-TOF, a size which is totally distinct from that of typically reported 33 kDa monomer from *P. aeruginosa*. The organic solvent stability experiment had demonstrated a stability pattern which completely opposed the rules laid out in previous reports in which activity stability and enhancement were observed in hydrophilic organic solvents such as DMSO, methanol, ethanol and 1-propanol. The high stability and enhancement of the enzyme in hydrophilic solvents were explained from the view of alteration in secondary structures. Elastinolytic activation and stability were observed in 25 and 50% of methanol, respectively, despite slight reduction in α-helical structure caused upon the addition of the solvent. Further characterization experiments had postulated great stability and enhancement of elastase strain K in broad range of temperatures, pHs, metal ions, surfactants, denaturing agents and substrate specificity, indicating its potential application in detergent formulation.

Keywords: *P. aeruginosa* strain K; organic solvent tolerant protease; dimerization; secondary structures

---

Table S1. Protein identification information for non- and TCA precipitated elastase strain K.

Protein name	Accession No.	Molecular weight (Da)	Protein score C.I%
Chain A, elastase of *P. aeruginosa* with an inhibitor	gi|52695999	33349.6	100
Hypothetical protein PaerPA_01004307 [*P. aeruginosa* PACS2]	gi|107103238	52322.7	100
Elastase precursor (EC 3.4.24.4)	gi|151212	53849.6	100
Elastase LasB [*P. aeruginosa* PAO1]	gi|15598919	53881.6	100
Elastase LasB [*P. aeruginosa* UCBPP-PA14]	gi|116051721	53795.5	100
Organic solvent tolerant elastase [*P. aeruginosa*]	gi|154127045	53732.6	100
Elastase LasB [*P. aeruginosa* 2192] $^{+}$	gi|194553400	53877.6	100
class 4 metalloprotease [*Chromobacterium violaceum* ATCC 12472] *	gi|34495512	53171.4	100
Elastase LasB [*P. aeruginosa* PA7]	gi|152985549	53940.7	100
class 4 metalloprotease [*Chromobacterium violaceum*] (gi|26419739) *	gi|26419739	53045.5	99.998

* Proteins identification derived from non-TCA precipitated sample only; $^{+}$ C.I% score of 71.775% from TCA precipitated sample.
